# Supplementary material for: Links between central CB1-receptor availability and peripheral endocannabinoids in patients with first episode psychosis
Source: NPJ Schizophr. 2020 Aug 26;6:21. doi: 10.1038/s41537-020-00110-7 (PMC7450081; doi:10.1038/s41537-020-00110-7)
Supplement: Supplementary file 1 — Supplementary material [file 41537_2020_110_MOESM1_ESM.pdf]

Links between central CB1-receptor availability and peripheral endocannabinoids in patients with first episode psychosis

Alex M. Dickens *et al.*

Supplementary Material

Supplementary Table 1. Tandem MS analysis of endocannabinoids. Information on quantification and selective reaction monitoring (SRM) transitions for identification.

Compound	Ionization	Ion Type	Retention Time	Internal Standard	SRM (amu)	CE (eV)
THC-COOH	Negative	Quantifier	1.23	THC-COOH d9	343.400 → 245.100	-32
THC-COOH	Negative	Qualifier	1.23	THC-COOH d9	343.400 → 299.000	-32
PEA	Positive	Quantifier	3.13	AEA-d8	300.400 → 62.100	19
PEA	Positive	Qualifier	3.13	AEA-d8	300.400 → 283.100	19
1-AG	Positive	Quantifier	3.24	2-AG d5	379.300 → 287.300	21
1-AG	Positive	Qualifier	3.24	2-AG d5	379.300 → 203.200	21
2-AG	Positive	Quantifier	2.97	2-AG d5	379.300 → 287.300	21
2-AG	Positive	Qualifier	2.97	2-AG d5	379.300 → 203.200	21
2-AGe*	Positive	Quantifier	3.23	2-AG d5	365.400 → 273.200	20
2-AGe*	Positive	Qualifier	3.23	2-AG d5	365.400 → 121.000	20
NADA*	Positive	Quantifier	2.69	NADA d8	440.300 → 137.100	30
NADA*	Positive	Qualifier	2.69	NADA d8	440.300 → 154.100	30
AEA	Positive	Quantifier	2.26	AEA-d8	348.300 → 62.100	32
AEA	Positive	Qualifier	2.26	AEA-d8	348.300 → 133.100	32
OEA	Positive	Quantifier	3.61	AEA-d8	326.300 → 62.100	20
OEA	Positive	Qualifier	3.61	AEA-d8	326.300 → 309.300	20
AA	Negative	Quantifier	4.48	AA-d8	303.300 → 259.100	-20
AA	Negative	Qualifier	4.48	AA-d8	303.300 → 58.900	-20
SEA	Positive	Quantifier	5.92	AEA-d8	328.400 → 62.100	23
SEA	Positive	Qualifier	5.92	AEA-d8	328.400 → 311.400	23

*Analyte below limit of detection in study samples.

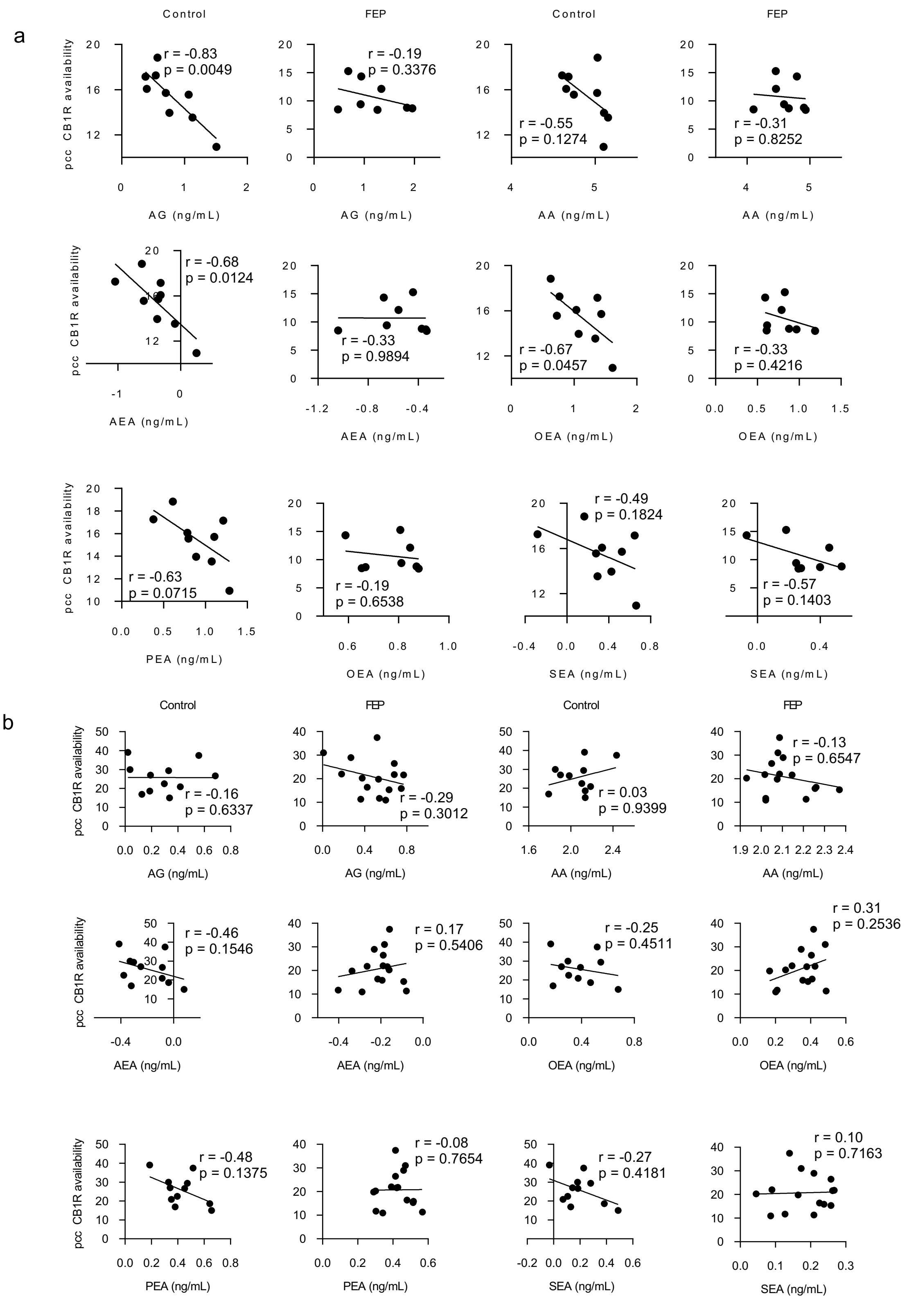

Supplementary Figure 1. CB1R availability in the posterior cingulate cortex (PCC) versus the irculating levels of endocannabinoids in Turku and London cohorts.

(a) Scatter plots fitted with a linear regression model of CB1R availability in the posterior cingulate cortex (PCC) versus the log-transformed circulating levels of endocannabinoids from the Turku cohort. (**b**) Scatter plots fitted with a linear regression model of CB1R availability in the posterior cingulate cortex (PCC) versus the log-transformed circulating levels of endocannabinoids from the London cohort. The line shows the linear model for each dataset.
