# Supplementary material for: Links between central CB1-receptor availability and peripheral endocannabinoids in patients with first episode psychosis
Source: NPJ Schizophr. 2020 Aug 26;6:21. doi: 10.1038/s41537-020-00110-7 (PMC7450081; doi:10.1038/s41537-020-00110-7)
Supplement: Supplementary file 2 — Reporting Summary [file 41537_2020_110_MOESM2_ESM.pdf]

nature research

Corresponding author(s): Dickens A, Oresic M

Last updated by author(s): Jun 24, 2020

Reporting Summary

Nature Research wishes to improve the reproducibility of the work that we publish. This form provides structure for consistency and transparency in reporting. For further information on Nature Research policies, see Authors & Referees and the Editorial Policy Checklist.

Statistics

For all statistical analyses, confirm that the following items are present in the figure legend, table legend, main text, or Methods section.

n/a	Confirmed	
☐	☒	The exact sample size (*n*) for each experimental group/condition, given as a discrete number and unit of measurement
☐	☒	A statement on whether measurements were taken from distinct samples or whether the same sample was measured repeatedly
☐	☒	The statistical test(s) used AND whether they are one- or two-sided<br>*Only common tests should be described solely by name; describe more complex techniques in the Methods section.*
☐	☒	A description of all covariates tested
☐	☒	A description of any assumptions or corrections, such as tests of normality and adjustment for multiple comparisons
☐	☒	A full description of the statistical parameters including central tendency (e.g. means) or other basic estimates (e.g. regression coefficient) AND variation (e.g. standard deviation) or associated estimates of uncertainty (e.g. confidence intervals)
☐	☒	For null hypothesis testing, the test statistic (e.g. *F*, *t*, *r*) with confidence intervals, effect sizes, degrees of freedom and *P* value noted<br>*Give P values as exact values whenever suitable.*
☒	☐	For Bayesian analysis, information on the choice of priors and Markov chain Monte Carlo settings
☒	☐	For hierarchical and complex designs, identification of the appropriate level for tests and full reporting of outcomes
☐	☒	Estimates of effect sizes (e.g. Cohen's *d*, Pearson's *r*), indicating how they were calculated

Our web collection on statistics for biologists contains articles on many of the points above.

Software and code

Policy information about availability of computer code

Data collection	Endocannabinoid measurements were performed using the instrument vendor software.
Data analysis	MATLAB scripts used for data analysis are available upon reasonable request.

For manuscripts utilizing custom algorithms or software that are central to the research but not yet described in published literature, software must be made available to editors/reviewers. We strongly encourage code deposition in a community repository (e.g. GitHub). See the Nature Research guidelines for submitting code & software for further information.

Data

Policy information about availability of data

All manuscripts must include a data availability statement. This statement should provide the following information, where applicable:

- Accession codes, unique identifiers, or web links for publicly available datasets
- A list of figures that have associated raw data
- A description of any restrictions on data availability

The data that support the findings of this study are available from the corresponding author upon reasonable request.

Field-specific reporting

Please select the one below that is the best fit for your research. If you are not sure, read the appropriate sections before making your selection.

☒ Life sciences ☐ Behavioural & social sciences ☐ Ecological, evolutionary & environmental sciences

For a reference copy of the document with all sections, see nature.com/documents/nr-reporting-summary-flat.pdf

Life sciences study design

All studies must disclose on these points even when the disclosure is negative.

Sample size	The patients at the two study sites were screened for eligibility according to the inclusion criteria,from January 5, 2015, through September 26, 2018.
Data exclusions	Some subjects were excluded due to increases in total arachidonoyl glycerol levels, caused by sample handling (as described in Methods).
Replication	The study includes separate analysis of two studies of similar study design, performed in Turku and London, respectively.
Randomization	The study includes two groups, first-episode psychosis patients and healthy controls.
Blinding	The investigators involved in the analysis of circulating endocannabinoids were blinded to the group allocation.

Reporting for specific materials, systems and methods

We require information from authors about some types of materials, experimental systems and methods used in many studies. Here, indicate whether each material, system or method listed is relevant to your study. If you are not sure if a list item applies to your research, read the appropriate section before selecting a response.

Materials & experimental systems

n/a	Involved in the study	
☒	☐	Antibodies
☒	☐	Eukaryotic cell lines
☒	☐	Palaeontology
☒	☐	Animals and other organisms
☐	☒	Human research participants
☒	☐	Clinical data

Methods

n/a	Involved in the study	
☒	☐	ChIP-seq
☒	☐	Flow cytometry
☒	☐	MRI-based neuroimaging

Human research participants

Policy information about studies involving human research participants

Population characteristics	Patients with FEP met the following inclusion criteria: (1) DSM-IV diagnosis of a psychotic disorder, determined by the Structured Clinical Interview of DSM-IV-TR Axis I Disorders–Patient Edition; (2) illness duration of less than 3 years; and (3) male sex. In Turku study, volunteers were taking antipsychotics and had diagnoses of affective or nonaffective psychosis.27 In London study, volunteers were medication naive or free of all pharmacological treatments for at least 6 months and had diagnoses of schizophrenia or schizoaffective disorder. Healthy volunteers had no current or lifetime history of an Axis I disorder as determined by the Structured Clinical Interview of DSM-IV-TR Axis I Disorders–Patient Edition and were matched by age (±3 years) and sex (male). Exclusion criteria for all volunteers were (1) current or lifetime history of substance abuse or dependence; (2) substance use within the last month; and (3) positive results for cannabis and other substances on screening toxicology tests.
Recruitment	For study in Turku, 14 patients were screened for eligibility, 13 were deemed eligible, and 8 were included in the study; and 25 healthy volunteers were screened for eligibility, 13 were deemed eligible, and 11 were included in the study. The overall number of patients and controls that were considered for inclusion was not recorded for study 1. For London study, clinical teams indicated that approximately 400 patients (3% of 125 000 patients seen during a 4-year period by clinical teams) were potentially eligible, 115 patients were examined for eligibility, and 106 patients were deemed eligible and included in the study. However, 66 of 106 patients (62.3%) were later withdrawn from the study owing to a loss of capacity to consent, hospital admission, or the commencement of antipsychotic treatment. A total of 40 patients were included in the study. We identified 300 potentially eligible healthy volunteers, of whom 40 were deemed eligible and were included in the study.
Ethics oversight	London - Camberwell St Giles Research Ethics Committee; The Ethics Committee, Turku University Hospital

Note that full information on the approval of the study protocol must also be provided in the manuscript.
